# Assessment of Physician Well-being, Part Two: Beyond Burnout

**DOI:** 10.5811/westjem.2019.1.39666

**Published:** 2019-02-28

**Authors:** Michelle D. Lall, Theodore J. Gaeta, Arlene S. Chung, Sneha A. Chinai, Manish Garg, Abbas Husain, Cara Kanter, Sorabh Khandelwal, Caitlin S. Rublee, Ramin R. Tabatabai, James Kimo Takayesu, Mohammad Zaher, Nadine T. Himelfarb

**Affiliations:** * Emory University School of Medicine, Department of Emergency Medicine, Atlanta, Georgia; † New York-Presbyterian Brooklyn Methodist Hospital, Department of Emergency Medicine, Brooklyn, New York; ‡ Weill Cornell Medicine, Department of Emergency Medicine in Clinical Medicine, New York, New York; § Maimonides Medical Center, Department of Emergency Medicine, Brooklyn, New York; ¶ University of Massachusetts Medical School, Department of Emergency Medicine, Worcester, Massachusetts; || Lewis Katz School of Medicine at Temple University, Department of Emergency Medicine, Philadelphia, Pennsylvania; # Temple University Hospital, Department of Emergency Medicine, Philadelphia, Pennsylvania; ** Staten Island University Hospital Zucker School of Medicine at Hofstra/Northwell, Department of Emergency Medicine, Staten Island, New York; †† The Ohio State University, Department of Emergency Medicine, Columbus, Ohio; ‡‡ Keck School of Medicine of USC, Department of Emergency Medicine, Los Angeles, California; §§ Harvard Medical School, Department of Emergency Medicine, Boston, Massachusetts; ¶¶ Prince Mohammad Bin AbdulAziz Hospital, Consultant of Emergency Medicine, Riyadh, Saudi Arabia; |||| Alpert Medical School of Brown University, Department of Emergency Medicine, Providence, Rhode Island

## Abstract

Part One of this two-article series reviews assessment tools to measure burnout and other negative states. Physician well-being goes beyond merely the absence of burnout. Transient episodes of burnout are to be expected. Measuring burnout alone is shortsighted. Well-being includes being challenged, thriving, and achieving success in various aspects of personal and professional life. In this second part of the series, we identify and describe assessment tools related to wellness, quality of life, resilience, coping skills, and other positive states.

## INTRODUCTION

In 2009, Shanafelt and colleagues proposed that “wellness goes beyond merely the absence of distress and includes being challenged, thriving, and achieving success in various aspects of personal and professional life.”^1^ Siedsma and Emle defined it as “the complex and multifaceted nature of physicians’ physical, mental and emotional health, and well-being.”^2^ The American College of Emergency Physicians (ACEP) proposed a multidimensional wellness model in 2016, the components of which are the following: occupational, emotional, physical, financial, spiritual, social, and intellectual. When viewing this model, it is clear how these areas are interconnected and critical in a person’s everyday life and that any approach to well-being must offer a holistic approach incorporating the different psychosocial aspects affecting the physician.^3^,^4^ Despite these and other frameworks, no clear consensus definition of well-being exists in the academic medical literature.^5^ Well-being is comprised of multiple variables including work-life balance, quality of life, resilience, mindfulness, coping strategies, and mood. In this review we explore the tools that assess the positive states of physician well-being.

The importance of physician well-being has now been universally recognized, with calls to action made by virtually every major medical society, including the American Medical Association, the Association of American Medical Colleges, and the Accreditation Council for Graduate Medical Education and in emergency medicine (EM) by the Council of Residency Directors (CORD), ACEP, and the Society for Academic Emergency Medicine. Whether wellness and well-being can be taught remains to be determined, and there is no standard for assessment or improvement. Numerous studies have looked at various aspects, but either due to small or specific sample size or confounding factors that lacked consideration, interpretation of and extrapolation of the results of these interventions should be done with caution.^6^

### Summary

Well-being is a complex and multifactorial topic. Accurate measurement is key to conducting needs assessments, developing appropriate interventions, and ongoing monitoring.^7^ There are numerous tools available for assessment. The goal of the Assessment Tools Workgroup, a sub-committee of the CORD Resilience Committee, was to research and summarize the various assessment tools available on burnout, well-being, resilience, and related factors and compile them as a collated resource. This is the second resource available in this series; it focuses on assessment tools to measure well-being, resilience, and other positive states. For assessment tools related to burnout and negative states, please refer to “The Assessment of Physician Well-being, Part One: Burnout and Other Negative States.”

## METHODS

The instruments included in this article are the result of a scoping review of English-language publications with abstracts indexed in PubMed, Web of Science, and MedEd Portal within the past 10 years. Searches were based on the main Medical Library Subject Heading (MeSH) terms “resilience,” “mindfulness,” “mood,” “personality,” “well-being,” “quality of life,” and “stress.” In addition to the search on the main term, subheadings included the following: measurement, assessment, evaluation, diagnosis, education, etiology, trends, derivation, validation, tool, instrument, scale, measure, survey, or questionnaire AND resident, residency, intern, internship, medical student, clerk, attending, physician and clinician. A complete listing of search terms can be found in Appendix 1. This search was augmented by reviewing article reference lists and performing further citation searches. We did not include instruments cited only in abstracts or as reports of meetings.

Abstractors performed a comprehensive review of the identified assessment tools. Details of all scales and where they can be found are presented in Table and Appendix 2. The tools identified as most relevant, accessible, and practical in evaluating emergency physician (EP) well-being were included for further review. The tools were selected by multiple abstractors. Abstractors worked in groups of two or three and focused on one subject (e.g., mindfulness or quality of life). Discrepancies between abstractors were reviewed by either the first, second, or last author on the manuscript. Consensus between at least two reviewers was required for an instrument to be included in this paper.

The primary inclusion criteria was use of the tool in a physician population in the medical literature. Exclusion criteria included tools that were not used in a physician population or were not cited in the medical literature relating to physicians more than two to three times. Tools that did not meet these two criteria are referenced in Appendix 2. The figure illustrates the search algorithm and tool selection process. The articles reviewed were organized by subcategory of the tool (e.g., mindfulness tools), then by individual tool, and finally, by the populations the tool had been used in.

A summary of the scale’s purpose, structure, and evidence of its psychometric properties were derived from the original source references. Due to the varied psychometric properties of each tool, abstractors relied on the reported validity and reliability from the source manuscripts. Where available, published cutoff scores are provided for guidance, although their validity or utility in other clinical or research contexts should not be assumed. Where psychometric properties were not explicitly described in the primary sources, potential users may need to check for any subsequent information pertaining to reliability and validity.

The following comments and discussions should be read in conjunction with the details reported in Table and Appendix 2, as well as with the recommendations provided at the end of the review.

## RESULTS

### Well-being Factors and Quality of Life Tools

While the definition of job burnout is relatively clear,^8^ well-being has been viewed through various domains^9^ and used interchangeably with quality of life (QOL).^10^ Higher perception of work-life imbalance negatively impacts work satisfaction and effect of work on QOL.^11^ Several authors investigating well-being in physicians used instruments initially intended for the general population or patients,^10^,^12^ while others derived instruments that specifically address the physician population.^13^

#### Physician Wellness Inventory

The Physician Wellness Inventory (PWI) is a measure of how happy and satisfied physicians are with their work. The PWI was piloted to assess attendings, residents and fellows from three academic centers in Michigan in 2010. The first and only study that used the PWI was performed in randomly selected full-time physician members of the American Academy of Family Physicians to assess the relationship between burnout and happiness. They found that career purpose was the strongest predictor of happiness.^14^ No other studies have evaluated the reliability and validity of this instrument. The major advantage of the PWI is that it was developed for physicians, taking into consideration their work settings and relationship to patients.

#### Physician Well-being Index

The authors at the Mayo Clinic School of Medicine developed the Physician Well-Being Index (PWBI) specifically for medical professionals, including resident and medical student versions.^13^,^15^,^16^ The purpose of the index is to stratify physician well-being in several important dimensions and to identify physicians whose degree of distress may negatively impact their practice (career satisfaction, intent to leave current position, medical errors). The seven-item survey includes domains of burnout, depression, stress, fatigue, and mental and physical QOL.

#### Quality of Life Linear Analog Self-assessment

The Quality of Life Linear Analog Self-Assessment (QOL LASA) scales have repeatedly been used in the literature to evaluate QOL in the cognitive, physical, emotional, social, or spiritual domains.^17^ In medical oncologists, high QOL LASA scores were associated with higher work satisfaction.^18^ One study employed the QOL LASA to measure the outcomes of a well-being initiative in a group of 40 internal medicine (IM) physicians who subsequently demonstrated a significant increase in QOL LASA scores post-intervention group.^60^ QOL LASA scores have been shown to be negatively correlated with self-perceived error reporting in several studies.^19^–^21^

#### Professional Quality of Life Scale

The Professional Quality of Life Scale (ProQOL) is the current iteration of several previously developed scales related to compassion fatigue including the Compassion Fatigue Scale, the Compassion Fatigue Self-Test, and the Compassion Satisfaction and Compassion Fatigue Test. ProQOL is a well-validated, 30-item scale that consists of three separate subscales: compassion satisfaction; burnout; and secondary traumatic stress. There are over 650 citations related to ProQOL, and its previous iterations in the medical literature. Key literature can be found here: http://www.proqol.org/uploads/ProQOL_Concise_2ndEd_12-2010.pdf. The ProQOL scale allows for monitoring of both the negative consequences (e.g., burnout and secondary traumatic stress) and protective qualities (e.g., compassion and satisfaction) of being in a caring profession.

#### Epworth Sleepiness Scale

Fatigue has long been linked to well-being and QOL. Sleep loss and fatigue have a significant negative impact on resident quality of life and perception of well-being.^22^ The Epworth Sleepiness Scale (ESS) is the most widely used tool to evaluate daytime sleepiness in a variety of populations and cultures. In multiple medical student studies, there were high rates of daytime sleepiness, higher levels of burnout, and academic performance.^23^–^28^ Increased sleepiness has been shown to be related to an increase in motor vehicle accidents among IM residents,^29^ with higher levels of stress and fatigue being independently associated with self-perceived medical errors.^30^

In a study with EM residents, sleep deprivation was found to significantly impact resident lives both personally and professionally with many social activities and meaningful personal pleasures being deferred or postponed during residency.^31^ Additionally, residents in that study reported that sleep loss and fatigue had a major impact on their ability to perform their work. While baseline characteristics have not been established and cross-specialty studies have not been done, one study of IM residents found that 23% had an abnormal ESS score.^32^

### Coping Tools: Resilience and Mindfulness

EPs are subjected to high-stress situations on a regular basis. Stress is not burnout but a natural response defined as a state of mental or emotional strain or tension resulting from adverse or very demanding circumstances. Reaction to stressors is highly individualized, and numerous emotional and physical disorders have been linked to stress. Understanding the response to stress can provide insight into specific behavioral modifications that can be used to cope with stress in more positive ways.

There are numerous types of coping mechanisms, some positive and associated with increased mindfulness and resilience, and some negative, which worsen symptoms of burnout. Resilience, too, is a unique and central component of well-being, identified as “the ability of an individual to respond to stress in a healthy, adaptive way such that personal goals are achieved at minimal psychological and physical cost.”^33^ It is increasingly recognized as a strategy that may reduce physician stress, particularly burnout, anxiety, and depression. In one study conducting semi-structured interviews with a variety of physicians, self-awareness, self-monitoring, and mindfulness-based, stress-reduction techniques were determined to be as effective as techniques to reduce the negative feelings of emotional distress and consequent rumination while enhancing a physician’s capacity for empathy.^34^ This notion of self-awareness and self-care is thought to be teachable and can be enhanced, as demonstrated by one pilot study involving family medicine residents.^35^

#### Connor Davidson Resilience Scale

The Connor Davidson Resilience Scale (CD-RISC) was developed for clinical practice for patients with mental health concerns, particularly post-traumatic stress disorder and anxiety, as a measure of stress and adaptability. The initial paper also states three potential uses: to explore the biologic aspects of resilience; to use in clinical practice in an effort to recognize resilient characteristics and evaluate responses to interventions; and as a screening tool for high-risk, high-stress activities or occupations.^36^ The initial intent was to use the scale with patients suffering from mental illness. The CD-RISC targets five factors: personal competence; trust/tolerance/strengthening effects of stress; acceptance of change and secure relationships; control; and spiritual influences. The tool has been shown to have convergent and discriminant validity and to be reliable in multiple nationalities and populations.^37^,^38^

While it is one of the most widely used instruments for resilience, the CD-RISC may have some limitations, specifically a “ceiling effect.” In other words, the scale’s lack of items to detect higher levels of resilience characteristics as variables and of its capacity to measure higher levels of resilience limits its usefulness in analyzing certain professions known for higher levels of resilience, and thus may be deficient.^39^

#### Perceived Stress Scale

The Perceived Stress Scale (PSS) measures the degree to which situations in one’s life are viewed as stressful. This tool was designed with the intent of creating a psychometrically sound global measure of perceived stress that could provide information on the relationship between stress and pathology.^40^ The PSS has been widely used across the globe and most frequently with university students and those attempting to stop smoking. There are multiple studies on resident physicians who have used the PSS as an assessment of their well-being. In several of these studies, perceived overall stress was strongly related to work hours and was found to affect physicians more than other healthcare professionals (e.g., nurses).^41^ Resident physicians who screen positive for burnout also have higher perceived levels of stress, a pattern similarly shown in faculty physicians.^42^,^43^ Resident physicians have also been noted to have higher perceived levels of stress than matched controls in the general population.^43^ In several studies with nurses and faculty physicians, implementation of a resiliency program has shown improvement in scores on the PSS.^44^–^46^

The PSS is a highly reliable and valid measure of stress in adults across multiple ethnicities. It provides individuals insight into their typical stress-response state. This awareness, in turn, may potentially increase mindfulness and be used to target relaxation behaviors to relieve stress. A potential limitation of the PSS is that the initial intent of the tool was to link stress to pathologic behavior, particularly tobacco abuse. However, many studies in the medical literature have used the PSS to assess patients pre- and post-intervention for multiple disease processes. If one considers burnout symptoms pathological insofar as they have been linked to issues such as substance abuse, medical error, and poor patient satisfaction, then this limitation is of debatable significance.

#### Coping Inventory to Stressful Situations

The Coping Inventory to Stressful Situations (CISS) measures three types of coping styles when one encounters a stressful or challenging situation: emotion-oriented; task-oriented; and avoidance-oriented coping. It also measures distraction and social diversion. The tool is aimed at determining an individual’s preferred coping style to provide a better understanding of the relationship between that individual’s coping style and his or her personality. The results can be used to help intervention planning for individuals in stressful situations. There is also a modified 21-item tool for specific social situations or interpersonal conflicts (CISS: Situation-Specific Coping Measure [CISS:SSCM]).

The CISS and CISS:SSCM are reliable measures of coping styles, demonstrating internal consistency, test-retest reliability, and item-remainder correlation. The CISS scales also have demonstrated construct validity as assessed by factor analysis in adults, undergraduates, and adolescents.^47^ Data from the study of medical students and physicians in practice have shown that task-oriented coping plays a role in reducing burnout symptoms, while emotion-oriented and avoidance-oriented coping may do the opposite. Therefore, the CISS may be a valuable tool in identifying individuals who may require additional training in specific coping strategies to improve their resilience and/or reduce their risk of burnout.^48^

In a study of 616 emergency department (ED) personnel, increased levels of burnout were associated with emotion-oriented coping while decreased levels were observed in those with task-oriented coping.^49^ This was also demonstrated in a study of 50 IM physicians.^50^ The CISS has also been used to study medical students, demonstrating a correlation of task-oriented coping with higher levels of emotional intelligence.^51^ Emotional intelligence – the ability to perceive, process, and regulate emotions effectively – is thought to be a strong predictor of resident well-being.^52^ Two other studies of medical students found that avoidance-oriented coping was associated with increased measure of fatigue and depressive symptoms.^53^,^54^

#### The Ways of Coping Checklist, the Ways of Coping (Revised), the Ways of Coping Scale

The Ways of Coping Scales (WCCL, CAPS, WAYS) identify two distinct, general types of coping: problem-focused and emotion-focused. Problem-focused coping is aimed at problem solving or doing something to alter the source of stress. Emotion-focused coping is aimed at reducing or managing the emotional distress that is associated with or cued by the situation. While most stressors will elicit both types of coping, problem-focused coping tends to predominate when the individual feels as if something constructive can be done, leading to engagement of the problem, and emotion-focused coping predominates when the individual believes the stressor is something that must be endured, leading to problem avoidance and disengagement.^55^,^56^ The Ways of Coping measures are not designed to assess coping traits and/or style. Each administration of the tool is aimed at understanding the coping processes an individual engages in a particular stressful encounter rather than attempting to define their coping style or traits.

The CAPS measure has potential benefit in prospectively identifying individuals with more emotion-focused coping strategies who may be at risk of burnout. Its main limitation is that this tool is situation-specific and does not reflect the complexity of the situations in medical practice nor encompass the entirety of an individual coping skillset. Its strength lies in making an individual aware of how he or she copes with different, specific, stressful situations and providing language around coping responses that may be more mindful, healthful, and productive for them, their team, and their patients.

#### Coping Orientation to Problems Experienced

The Coping Orientation to Problems Experienced (COPE) Inventory and, more recently, the Brief COPE were designed to assess the different ways in which people respond to stress. This tool looks at many dimensions of coping, including both functional and dysfunctional responses. These dimensions include the following: active coping; planning; suppression of competing activities; restraint coping; seeking social support for instrumental reasons; seeking social support for emotional reasons; focusing on and venting emotions; behavioral disengagement; positive reinterpretation and growth; denial, acceptance; and turning to religion.^59^,^60^

The COPE Inventory was validated in a population of almost 1,000 undergraduate students in its final iteration. The authors state there is no such thing as an “overall” score on this measure and do not recommend a particular way of generating a dominant coping style for a given person. Instead, they advocate looking at each scale separately to see how it relates to the other variables. Thus, this tool may help with insight into coping response and personal reflection.

The Brief COPE has been used in studies involving IM and EM residents. In the study with IM residents, those residents who employed the strategies of acceptance, active coping, and positive reframing had lower emotional exhaustion and depersonalization, suggesting that residents who place a high priority on healthful relationships, engage in spiritual activities, and practice humility may have important coping mechanisms that mitigate burnout.^56^,^58^,^59^ Residents who employed denial, disengagement, self-blame, and humor were found to have higher emotional exhaustion and depersonalization. Disengagement and venting were found to be negatively correlated with personal accomplishment.

These tools are relatively short, free, and easy to use. They provide individuals with insight into their typical coping responses, thereby increasing mindfulness. Although used in physician populations, they have not been validated specifically in physician populations.

### Mood and Personality Tools

Personality typing is a psychological concept popularized in the 1940s. Conceptually, it is founded on the notion that individuals favor certain psychological preferences and that personality traits affect how they perceive and interact with their environments. The ED is a unique medical environment where there is great emphasis on leadership, communication, and teamwork. The application of personality and mood assessment instruments may provide useful information about individual ED providers and create a more dynamic, efficient, and sound working environment.

#### Myers-Briggs Type Indicator

The Myers-Briggs Type Indicator (MBTI) is the most widely used of the personality assessment tools. This introspective, self-assessment tool separates people into four dichotomies that each focus on a particular aspect of information processing: extraversion/introversion; sensing/intuition; thinking/feeling; and judging/perceiving. In the medical community, the MBTI has been studied in medical students, dental students, and resident and attending physician populations, primarily among surgeons and anesthesiologists. Many researchers have applied the MBTI to assess for personality patterns among different specialties and identify personalities at increased risk for burnout.^61^–^64^ While the MBTI is the oldest and most well-studied in the physician population, it has yet to be shown how MBTI results can be implemented to benefit individual practitioners and the work environment.

#### Profile of Mood States

The Profile of Mood States (POMS) is a self-report, psychological rating scale that assesses multiple dimensions of mood over a distinct period of time. Such mood states include the following: anger-hostility, confusion-bewilderment, depression-dejection, fatigue-inertia, tension-anxiety, vigor-activity, and friendliness. The dynamic nature of the assessment may allow for real-time evaluation for risks of burnout and second-victim syndrome following an unforeseen or unfavorable outcome.

While the tool has been used in several physician populations,^65^–^71^ it is not well validated in the medical field.^72^–^76^ The enthusiasm of IM interns was found to give way to sustained depression, anger, and fatigue at the end of internship.^67^ A study of early-career physicians showed that acute sleep deprivation secondary to long call hours negatively affected mood.^65^ In one study of IM residents, mood disturbances were identified as common, and the decline in empathic concern was specifically found to persist throughout training unlike other mood disturbances that were no longer present by the end of residency.^67^

#### Thomas-Kilmann Conflict Mode Instrument

The Thomas-Kilmann Conflict Mode Instrument (TKI) is a self-assessment tool that identifies individual conflict-handling styles, which are categorized into five “modes:” competing, collaborating, compromising, avoiding, and accommodating. Conflict is inevitable in any team-based field due to personality and work-style differences. EPs manage the expectations and reactions of patients in crisis. Patient-centered care requires collaboration between the clinicians, patients, family, and other providers, which may lead to another source of conflict.

The TKI has been validated in the medical population including nurses, residents, board-certified physicians, hospital administrators, and program directors, although not specifically among emergency clinicians.^77^–^80^ In studies of resident physicians, there is a tendency for higher levels of accountability and successful execution of administrative tasks in individuals with collaborating or competing conflict modes.^78^,^79^ Identifying individual conflict-management styles, the TKI can help provide insight to EPs regarding their strengths and potential weakness in dealing with conflict, which may ultimately help them become better leaders in the department and better team players.

## LIMITATIONS

There are an overwhelming number of assessment tools available in the literature that can be used to measure the different components of physician well-being. While our literature search was methodical and broad, we acknowledge that we may have missed some key assessment tools. At times, a single author determined the inclusion eligibility of the tools identified in our literature search strategy. However, consensus between at least two reviewers was required for an instrument to be included in this paper.

Assessment tools must be suitable for and validated in the population of interest. A majority of the tools we found have been used in a physician population but have never been validated in this population. Many of the tools have been designed for and validated in special populations; however, their applicability, reliability, and validity in a physician population is not clearly demonstrated in the medical literature. In the absence of independent validation, the results of these tools should be interpreted with caution.

Physician well-being is multifactorial, and it is difficult to purely divide these components by topic or sub-category as they have a complex interplay with one another. We have reviewed the tools based on the well-being topics that were most commonly found in the medical literature and that were of highest potential value. Very few tools exist that were either designed for use in a physician population or have been validated in physicians. We have highlighted the tools from each topic that are most relevant for use in assessing an EP population.

## CONCLUSION

Physician well-being is a complicated topic, and there is no standardized approach for assessing it. We provide a framework of the assessment tools that can be used to evaluate the positive states of physician well-being. The assessment tools reviewed vary in the topic assessed, cost, length, and applicability to a physician population. This manuscript is intended to provide the reader with several available options for evaluating different components of physician well-being. It is at the discretion of the reader to determine which tool would be most appropriate for the outcome he or she is trying to measure. There is great opportunity for the development of new tools and validation of those that are already in use.

There is clearly much to be done in the development of resources to mitigate burnout, foster resilience, and improve well-being. When undertaking an assessment of physician well-being, it is of critical importance to understand what you want to assess and to ensure you have selected the best tool to assess it. We hope this manuscript gives emergency physicians a starting point to evaluate their own well-being and the well-being of their peers, trainees, and students.

## Supplementary Material





## Figures and Tables

**Figure 1:**
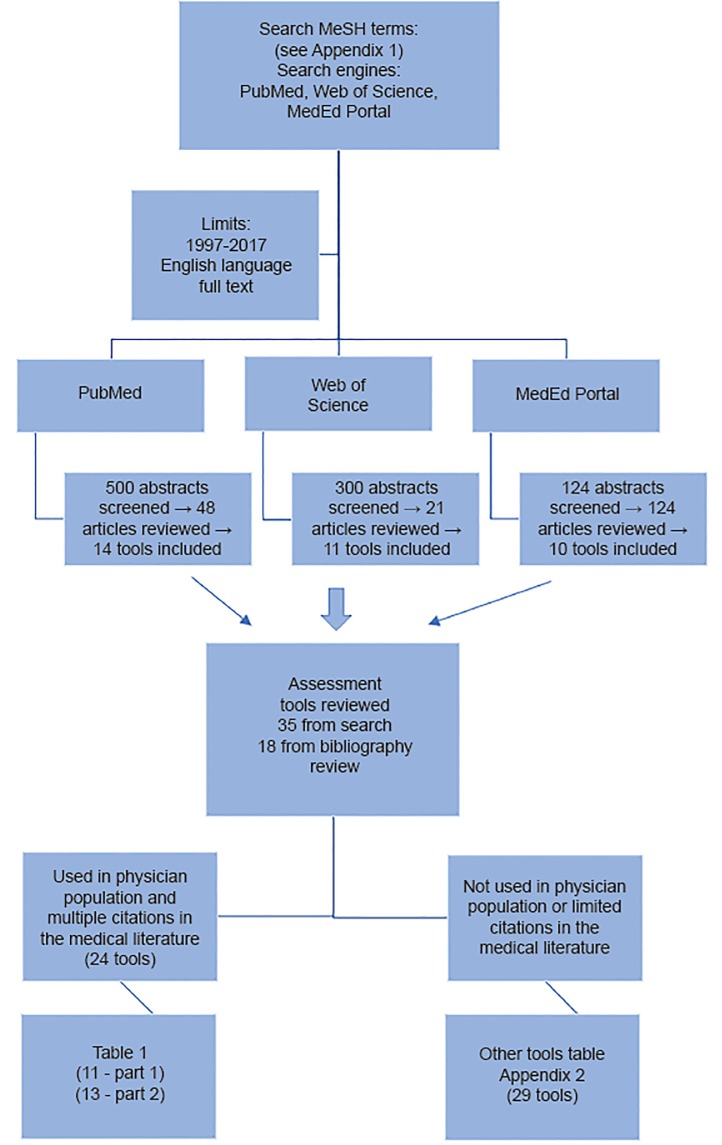
Flow diagram of literature search algorithm and assessment tool selection.

**Table:** Positive state assessment tools.

Name of instrument	Brief description	Number of items	Time to complete	Cost	Where to find it	Notes	Pros	Cons
Well-being and quality of life								
Physician Wellness Inventory	It has three scales: career purpose, cognitive flexibility and distress.	14 items	Two minutes	free	www.promoteyourwellness.com/PWI.docx	There are only two published studies using this instrument	Free Developed for physicians	Limited studies, need more data on reliability and validity
Physician Well Being Index (PWBI)	Used to: 1) stratify physician well-being in several important dimensions; and 2) identify physicians whose degree of distress may negatively impact their practice.	Seven Items	< Five minutes	Free for individuals Organizations: $10k license and $5k yearly fee	https://www.mededwebs.com/employee-well-being-index	Designed to measure burnout, provide valuable resources when people them the most, and track progress over time to promote self-awareness.	Short Externally validated Can be used for self-screening Provides self-directed learning resources	Costly More useful for screening than detailed testing
Quality of Life Linear Analog Scale Assessment (LASA)	LASA includes five simple items, each of which targets a specific domain of quality of life.	Five items	< Five minutes	Free	http://www.jpsmjournal.com/article/S0885-3924(07)00463-0/pdf	Specific domains include physical well-being (i.e., fatigue, activity level), emotional well-being (i.e., depression, anxiety, stress), spiritual well-being (i.e., sense of meaning, relationship with God), and intellectual well-being (i.e., ability to think clearly, concentrate).	Short Accessible Validated in multiple physician populations	Multiple forms exist
Professional Quality of Life Scale (ProQOL)	Self-report measure that asks the respondent to reflect on his or her experiences at work as a human service provider, both positive and negative, in the past 30 days.	30 items	5–10 minutes	Free, must credit the author	http://www.proqol.org/Home_Page.php	The ProQOL consists of three separate subscales: Compassion Satisfaction, Burnout, and Secondary Traumatic Stress. Standardized scores exist for all three (< 23 = low, 23–41 = average, > 41 = high). No composite score is available. It is recommended to complete the measure in its entirety rather than separate the questions into separate tests divided by subscale.	Free Validated Good reliability	Indirect measure of “wellness”
Epworth Sleepiness Scale (ESS)	Self-reported measure of how easily a person can fall asleep in different situations	Eight items	One minute	Free for individual use (Need a license for corporate use)	http://epworthsleepinessscale.com/about-the-ess/	The ESS specifically distinguishes reports of dozing behavior from feelings of fatigue and drowsiness/sleepiness	Free Quick and easy to use	Subjective Risk for bias
Resilience and mindfulness								
Connor Davidson Resilience Scale (CD-RISC)	Used for clinical practice as a measure of stress and adaptability. Also used to evaluate response to clinical interventions.	25 items	5–10 minutes	Need agreement from authors with small fee Cost is dependent on type and extent of use	http://www.connordavidson-resiliencescale.com/index.php	The scale has been developed and tested as a measure of degree of resilience. The scale also has promise as a method to screen people for high, intermediate or low resilience.	Well validated	Small fee. Initial intent to use on patients with mental illness. Limit use in physicians.
Perceived Stress Scale (PSS)	Used to measure the perception of stress; measure of the degree to which situations in one’s life are appraised as stressful; items are designed to tap how unpredictable, uncontrollable and overloaded respondents find their lives; direct queries of current experienced stress	14 items	10–15 minutes	Free	http://www.psy.cmu.edu/~scohen/scales.html	A psychometrically sound global measure of perceived stress that could provide valuable information about the relationship between stress and pathology (correlations between high perceived stress and burnout).	Free Short Easy to use	Not validated in health care providers
Coping Inventory for Stressful Situations (CISS)	The CISS measures three types of coping styles: task-oriented coping, emotion-oriented coping, and avoidance-oriented coping. It helps you determine the preferred coping style.	48 items	10 minutes	CISS Manual = $57 Quik Score Form (25/pkg)=$60	http://www.mhs.com	Offers precision in predicting preferred coping styles, and contributes to understanding the differential relationships between coping styles and other personality variables.	Reliable and valid Tests the interaction of stress, anxiety, and coping	Cost Not validated in a physician population
Ways of Coping Scale (WAYS)	The Ways of Coping Questionnaire is a 66-item instrument containing a wide range of thoughts and acts that people use to deal with the internal and/or external demands of specific stressful encounters.	66 items	10 minutes	$50 for the manual $2.50/license (50 surveys minimum)	http://www.mindgarden.com/158-ways-of-coping-questionnaire	An assessment of coping in relation to a specific stressful encounter. Not designed to be used as an assessment of coping styles or traits.	Well validated	Cost Length of instrument Not validated in a physician population.
The COPE Inventory (brief)	The COPE Inventory is a multidimensional coping inventory to assess the different ways in which people respond to stress.	28 items	15 minutes	Free	www.psy.miami.edu/faculty/ccarver/sclBrCOPE.html	Five scales (of four items each) measure conceptually distinct aspects of problem-focused coping (active coping, planning, suppression of competing activities, restraint coping, seeking of instrumental social support) Provides individual’s insight into their typical coping response leading to increased mindfulness	Free Easy to use	Not validated in a physician population. Intended use is to provide insight into a typical coping response not a coping style.
Mood and personality								
Myers-Briggs Type Indicator (MBTI)	Introspective self-assessment tool that identifies psychological preferences in how people interact with their environments and make decisions.	93 items	15 minutes	$49.95 per user	https://www.cpp.com/products/mbti/index.aspx	Provides insight into an individual’s personality traits, can help us identify weaknesses and be better communicators and decision makers	Widely used and highly regarded Validated Good reliability	Cost
Profile of Mood States (POMS 2)	Self-report psychological rating scale use to assess transient, distinct mood states. Measures multiple dimensions of mood over a distinct period of time which include: Anger-Hostility, Confusion-Bewilderment, Depression-Dejection, Fatigue-Inertia, Tension-Anxiety, Vigor-Activity and Friendliness.	Full version: 65 items Short version: 35 items	Full version: 10 minutes Short version: five minutes	Manual $92, and Single full or short form $3.50	https://ecom.mhs.com(S(4sbwc3qmfsjjpo454qllycuj))/inventory.aspx?gr=cli&prod=poms2&id=pricing&RptGrpID=pmr	Provides insight into an individual’s current mood state and how that may affect their performance at work and interaction with others.	Allows for real-time assessment of risks for burnout, second victim syndrome, etc.	Cost Not well validate in physician population
Thomas-Kilmann Conflict Mode Instrument (TKI)	Self-assessment tool that identifies individual conflict-handling styles, which are categorized into 5 “modes”: competing, collaborating, compromising, avoiding, and accommodating	30 item	15 minutes	$18.95 each, $179 pack of 10	https://www.cpp.com/en/tkiitems.aspx?ic=4813	Provides a pragmatic, situational approach to conflict resolution, change management, leadership development, and communication	Relevant Validated in physician populations	Cost

## References

[1] Myers MF (2001). The well-being of physician relationships. West J Med.

[2] Siedsma M, Emlet L (2015). Physician burnout: can we make a difference together?. Crit Care.

[3] Kaplan JA, Manfredi RA Emergency Medicine Wellness Week 2016 to focus on self-care for emergency physicians.

[4] Eckleberry-Hunt J, Van Dyke A, Lick D (2009). Changing the conversation from burnout to wellness: physician well-being in residency training Pprograms. J Grad Med Educ.

[5] Eckleberry-Hunt J, Lick D, Boura J (2009). An exploratory study of resident burnout and wellness. Acad Med.

[6] Williams D, Tricomi J, Gupta J (2015). Efficacy of burnout interventions in the medical education pipeline. Acad Psychiatry.

[7] Dyrbye LN, Trockel M, Frank E (2017). Development of a research agenda to identify evidence-based strategies to improve physician wellness and reduce burnout. Ann Intern Med.

[8] Maslach C, Schaufeli WB, Leiter MP (2001). Job burnout. Annu Rev Psychol.

[9] Kiefer RA (2008). An integrative review of the concept of well-being. Holist Nurs Pract.

[10] West CP, Shanafelt TD, Cook DA (2010). Lack of association between resident doctors’ well-being and medical knowledge. Med Educ.

[11] Mahmood S, Jackson R, Zhao YD (2015). Assessment of work-life balance of resident physicians. Am J Med Sci.

[12] Kassam A, Horton J, Shoimer I (2015). Predictors of well-being in resident physicians: a descriptive and psychometric study. J Grad Med Educ.

[13] Dyrbye LN, Satele D, Sloan J, Shanafelt TD (2014). Ability of the physician well-being index to identify residents in distress. J Grad Med Educ.

[14] Eckleberry-Hunt J, Kirkpatrick H, Taku K (2016). Relation between physicians’ work lives and happiness. South Med J.

[15] Dyrbye LN, Satele D, Sloan J (2013). Utility of a brief screening tool to identify physicians in distress. J Gen Intern Med.

[16] Dyrbye LN, Szydlo DW, Downing SM (2010). Development and preliminary psychometric properties of a well-being index for medical students. BMC Med Educ.

[17] Priestman TJ, Baum M (1976). Evaluation of quality of life in patients receiving treatment for advanced breast cancer. Lancet.

[18] Shanafelt TD, Novotny P, Johnson ME (2005). The well-being and personal wellness promotion strategies of medical oncologists in the North Central Cancer Treatment Group. Oncology.

[19] West CP, Tan AD, Habermann TM (2009). Association of resident fatigue and distress with perceived medical errors. JAMA.

[20] West CP, Huschka MM, Novotny PJ (2006). Association of perceived medical errors with resident distress and empathy: a prospective longitudinal study. JAMA.

[21] Kang EK, Lihm HS, Kong EH (2013). Association of intern and resident burnout with self-reported medical errors. Korean J Fam Med.

[22] Min AA, Sbarra DA, Keim SM (2015). Sleep disturbances predict prospective declines in residents’ psychological well-being. Med Educ Online.

[23] Pagnin D, de Queiroz V, Carvalho YT (2014). The relation between burnout and sleep disorders in medical students. Acad Psychiatry.

[24] Machado-Duque ME, Echeverri Chabur JE, Machado-Alba JE (2015). Excessive daytime sleepiness, poor quality sleep, and low academic performance in medical students. Rev Colomb Psiquiatr.

[25] Bahammam AS, Alaseem AM, Alzakri AA (2012). The relationship between sleep and wake habits and academic performance in medical students: a cross-sectional study. BMC Med Educ.

[26] Rodrigues RN, Viegas CA, Abreu E, Silva AA (2002). Daytime sleepiness and academic performance in medical students. Arq Neuropsiquiatr.

[27] Abdulghani HM, Alrowais NA, Bin-Saad NS (2012). Sleep disorder among medical students: relationship to their academic performance. Med Teach.

[28] Pikovsky O, Oron M, Shiyovich A (2013). The impact of sleep deprivation on sleepiness, risk factors and professional performance in medical residents. Isr Med Assoc J.

[29] Fruchtman Y, Moser AM, Perry ZH (2011). Fatigue in medical residents--lessons to be learned. Med Lav.

[30] West CP, Tan AD, Habermann TM (2009). Association of resident fatigue and distress with perceived medical errors. JAMA.

[31] Papp KK, Stoller EP, Sage P (2004). The effects of sleep loss and fatigue on resident-physicians: a multi-institutional, mixed-method study. Acad Med.

[32] Chen I, Vorona R, Chiu R, Ware JC (2008). A survey of subjective sleepiness and consequences in attending physicians. Behav Sleep Med.

[33] Epstein RM, Krasner MS (2013). Physician resilience: what is means, why it matters, and how to promote it. Acad Med.

[34] Zwack J, Schweitzer J (2013). If every fifth physician is affected by burnout, what about the other four? Resilience strategies of experienced physicians. Acad Med.

[35] Brennan J, McGrady A (2015). Designing and implementing a resiliency program for family medicine residents. Int J Psychiatry Med.

[36] Connor KM, Davidson JR (2003). Development of a new resilience scale: the Connor-Davidson Resilience Scale (CD-RISC). Depress Anxiety.

[37] Campbell-Sills L, Stein MB (2007). Psychometric analysis and refinement of the Connor–Davidson Resilience Scale (CD-RISC): validation of a 10-item measure of resilience. J Trauma Stress.

[38] Campbell-Sills L, Forde DR, Stein MB (2009). Demographic and childhood environmental predictors of resilience in a community sample. J Psychiatr Res.

[39] Arias Gonzalez VB, Crespo Sierra MT, Arias Martinez B (2015). An in-depth psychometric analysis of the Connor-Davidson Resilience Scale: calibration with Rasch-Andrich model. Health Qual Life Outcomes.

[40] Cohen S, Kamarck T, Mermelstein R (1983). A global measure of perceived stress. J Health Soc Behav.

[41] Chaukos D, Chad-Friedman E, Mehta DH (2017). Risk and resilience factors associated with resident burnout. Acad Psychiatry.

[42] Swami MK, Mathur DM, Pushp BK (2013). Emotional intelligence, perceived stress and burnout among resident doctors: an assessment of the relationship. Natl Med J India.

[43] Hutchison TA, Haase S, French S (2014). Stress, burnout, and coping among emergency physicians at a major hospital in Kingston, Jamaica. West Indian Med J.

[44] Waldman SV, Diez JC, Arazi HC (2009). Burnout, perceived stress, and depression among cardiology residents in Argentina. Acad Psychiatry.

[45] Deible S, Fiorvanti M, Tarantino B (2015). Implementation of an integrative coping and resiliency program for nurses. Global Adv Health Med.

[46] Sood A, Prasad K, Schroeder D (2011). Stress management and resilience training among Department of Medicine faculty: a pilot randomized clinical trial. J Gen Intern Med.

[47] Endler N, Parker JDA Coping Inventory for Stressful Situations.

[48] Endler NS, Parker JD, Butcher JN (1993). A factor analytic study of coping styles and the MMPI-2 content scales. J Clin Psychol.

[49] Howlett M, Doody K, Murray J (2015). Burnout in emergency department healthcare professionals is associated with coping style: a cross-sectional survey. Emerg Med J.

[50] Kwarta P, Pietrzak J, Miśkowiec D (2016). Personality traits and styles of coping with stress in physicians. Pol Merkur Lekarski.

[51] Wons A, Bargiel-Matusiewicz K (2011). The emotional intelligence and coping with stress among medical students. Wiad Lek.

[52] Lin DT, Liebert CA, Tran J (2016). Emotional intelligence as a predictor of resident well-being. J Am Coll Surg.

[53] Tanaka M, Fukada S, Mizuno K (2009). Stress and coping styles are associated with severe fatigue in medical students. Behav Med.

[54] Thompson G, McBride RB, Hosford CC (2016). Resilience among medical students: the role of coping style and social support. Teach Learn Med.

[55] Folkman S, Lazarus RS, Dunkel-Schetter C (1986). Dynamics of a stressful encounter: cognitive appraisal, coping, and encounter outcomes. J Pers Soc Psychol.

[56] Folkman S, Lazarus RS (1985). If it changes it must be a process: study of emotion and coping during three stages of a college examination. J Pers Soc Psychol.

[57] Howlett M, Doody K, Murray J (2015). Burnout in emergency department healthcare professionals is associated with coping style: a cross-sectional survey. Emerg Med J.

[58] Popa F, Arafat R, Purcărea VL (2010). Occupational burnout levels in emergency medicine--a stage 2 nationwide study and analysis. J Med Life.

[59] Carver CS, Scheier MF, Weintraub JK (1989). Assessing coping strategies: a theoretically based approach. J Pers Soc Psychol.

[60] Carver CS (1997). You want to measure coping but your protocol’s too long: consider the brief COPE. Int J Behav Med.

[61] Boyd R, Brown T (2005). Pilot study of Myers Briggs Type Indicator personality profiling in emergency department senior medical staff. Emerg Med Australas.

[62] Eicke FJ, Blake G, Replogle W (1993). A comparative view of the Myers-Briggs Type Indicator. Fam Med.

[63] Lemkau JP, Purdy RR, Rafferty JP (1988). Correlates of burnout among family practice residents. J Med Educ.

[64] Swanson JA, Antonoff MB, D’Cunha J (2010). Personality profiling of the modern surgical trainee: insights into generation X. J Surg Educ.

[65] Wali SO, Qutah K, Abushanab L (2013). Effect of on-call-related sleep deprivation on physicians’ mood and alertness. Ann Thorac Med.

[66] Smith-Coggins R, Howard SK, Mac DT (2006). Improving alertness and performance in emergency department physicians and nurses: the use of planned naps. Ann Emerg Med.

[67] Bellini LM, Shea JA (2005). Mood change and empathy decline persist during three years of internal medicine training. Acad Med.

[68] Bellini LM, Baime M, Shea JA (2002). Variation of mood and empathy during internship. JAMA.

[69] Hainer BL, Palesch Y (1998). Symptoms of depression in residents: a South Carolina Family Practice Research Consortium study. Acad Med.

[70] Wright SW, Lawrence LM, Wrenn KD (1998). Randomized clinical trial of melatonin after night-shift work: efficacy and neuropsychologic effects. Ann Emerg Med.

[71] Uliana RL, Hubbell FA, Wyle FA (1984). Mood changes during the internship. J Med Educ.

[72] Pollock V, Cho DW, Reker D (1979). Profile of Mood States: the factors and their physiological correlates. J Nerv Ment Dis.

[73] Berger BG, Motl RW (2008). Exercise and mood: A selective review and synthesis of research employing the Profile of Mood States. J Appl Sport Psychol.

[74] Nyenhuis DL, Yamamoto C, Luchetta T (1999). Adult and geriatric normative data and validation of the Profile of Mood States. J Clin Psychol.

[75] Spielberger CD (1972). Review of Profile of Mood States. Prof Psychol.

[76] Boyl GJ (1987). A cross-validation of the factor structure of the Profile of Mood States: Were the factors correctly identified in the first instance?. Psychol Rep.

[77] Ogunyemi D, Tangchitnob E, Mahler Y (2011). Conflict styles in a cohort of graduate medical education administrators, residents, and board-certified physicians. J Grad Med Educ.

[78] Ogunyemi D, Eno M, Rad S (2010). Evaluating professionalism, practice-based learning and improvement, and systems-based practice: utilization of a compliance form and correlation with conflict styles. J Grad Med Educ.

[79] Ogunyemi D, Fong S, Elmore G (2010). The associations between residents’ behavior and the Thomas-Kilmann Conflict MODE Instrument. J Grad Med Educ.

[80] Sportsman S, Hamilton P (2007). Conflict management styles in the health professions. J Prof Nurs.

